# Synergistic Improvement in the Thermal Conductivity of Hybrid Boron Nitride Nanotube/Nanosheet Epoxy Composites

**DOI:** 10.1021/acsanm.4c01646

**Published:** 2024-05-20

**Authors:** Rajeshkumar Mohanraman, Pietro Steiner, Coskun Kocabas, Ian A. Kinloch, Mark A. Bissett

**Affiliations:** Department of Materials, Henry Royce Institute, National Graphene Institute, University of Manchester, Manchester M13 9PL, U.K.

**Keywords:** thermal conductivity, nanocomposite, h-BN, boron nitride nanotube, epoxy, nanomaterial, thermal management

## Abstract

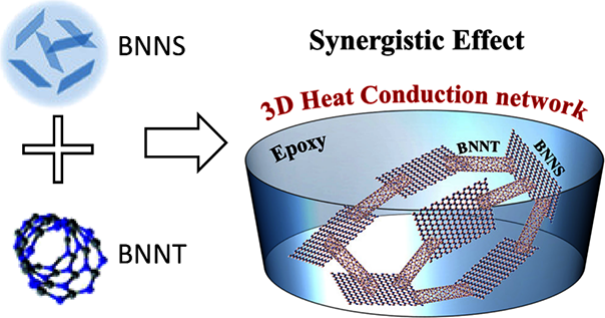

Epoxy composites with excellent thermal properties are highly promising for thermal management applications in modern electronic devices. In this work, we report the enhancement of the thermal conductivity of two different nanocomposites, using epoxy resins LY564 (epoxy 1) and LY5052 (epoxy 2), by incorporating multiwalled boron nitride nanotubes (BNNT) and boron nitride nanosheets (BNNS) as fillers. The synergistic interaction between the 1D BNNT and 2D BNNS allows for improved thermal conductivity *via* several different mechanisms. The highest thermal conductivity was measured at a loading of 1/30 wt % of BNNT/BNNS, resulting in values of 2.6 and 3.4 Wm^–1^ K^–1^, respectively, for each epoxy matrix. This improvement is attributed to the formation of a three-dimensional heat flow path formed through intercalation of the nanotubes between the BNNS. The thermal conductivity of the epoxy 1 and 2 nanocomposites improved by 940 and 1500%, respectively, making them suitable as thermal interface materials in electronic applications requiring electrical resistivity.

## Introduction

The high level of integration and increasing power density of electronic devices can lead to heat accumulation, which can negatively impact their performance and lifespan.^1,2^ Thermal interface materials (TIMs) are essential to manage this and are used to transfer heat from a heat source to a heat sink.^3,4^ Epoxy resins are commonly used in microelectronics because of their ease of use, excellent chemical stability, and low cost, but they often have low thermal conductivity ranging from 0.1 to 0.5 Wm^–1^ K^–1^ at room temperature.^5,6^ Despite the widespread use of low-cost ceramic fillers such as alumina (Al_2_O_3_),^7^ silicon nitride (Si_3_N_4_),^8^ aluminum nitride (AlN),^9^ and silicon carbide (SiC)^10^ as thermally conductive fillers, the resulting epoxy composites still demonstrate a low thermal conductivity, which remains below 2.0 Wm^–1^ K^–1^.

Hexagonal boron nitride (hBN) has a structure similar to graphene and has high thermal conductivity, but has the added benefit of electrical insulation, and also chemical stability.^11−14^ It is often used as a filler in polymer-based composites to improve the thermal conductivity. h-BN in the form of 2D nanosheets and 1D nanotubes also offers a promising alternative as they not only have similar thermal conductivity to carbon nanotubes (CNTs) and graphene but also have a larger bandgap (4–6 eV), making them suitable for electronic devices that require both electrical insulation and high thermal conductivity.^13^ Previous studies have reported that boron nitride nanotubes (BNNTs) had a high aspect ratio and high thermal conductivity (∼200 to 300 Wm^–1^ K^–1^),^15^ making them a suitable filler. Theoretical work has predicted that the thermal conductivity of a single-walled BNNT may even surpass that of CNTs.^16,17^ Therefore, this study designs a 3D filling strategy of BNNS/BNNT/epoxy composites to improve their thermal conductivity and examine the synergistic effect on the thermal conductivity of the hybrid composite. Small amounts of BNNTs are added to BNNS/epoxy composites by the speed mixing process, and the effect on thermal conductivity is evaluated by using laser diffusivity and novel modulated infrared (IR) thermoreflectance techniques. The method of creating these hybrid nanofiller-epoxy composites is simple and ecofriendly and can be easily scaled up, making them a potential option for use as TIMs in the next generation of electronic devices.

## Materials and Methods

### Materials

Few-layer hexagonal boron nitride (h-BN) nanoplatelet powder (particle size ∼5 μm) and graphene powder (particle size ∼5 μm) were purchased from Versarien Ltd., UK. Boron nitride nanotube (BNNT) powder with a purity of 90 wt % was purchased from Naieel Technology, South Korea. Araldite LY564 (bisphenol-A, epoxy resin)/Aradur 2954 (cycloaliphatic polyamine, hardener) and Araldite LY5052 (components: epoxy phenol novolac and 1,4-butanediol diglycidyl ether)/Aradur hardener HY5052 (components: isophorone diamine and cycloaliphatic diamine) were purchased from Huntsman Advanced Materials, USA.

### Fabrication of 3D Fill BN/Epoxy Composites

**Figure 1:**
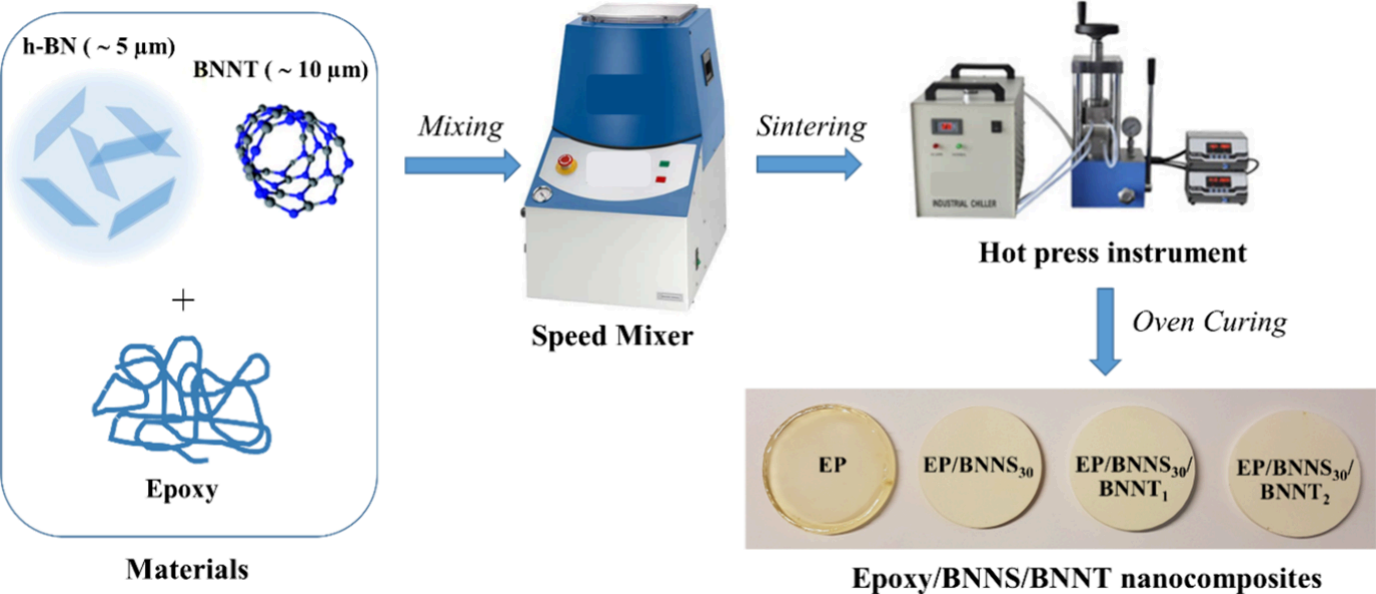
Schematic depicting the formation process of the 3D BN/epoxy composites.

The production process of the epoxy/BNNS/BNNT composites is illustrated in Figure 1. For the fabrication of the epoxy composites, we used a weight ratio of 100:35 for Araldite LY564 (epoxy 1)/Aradur hardener 2954 and a weight ratio of 100:38 for the Araldite LY5052 (epoxy 2)/Aradur hardener HY5052. The composites were created by blending and curing the fillers (BNNS and BNNT powders) with epoxy with loadings ranging from 0 to 30 wt % for BNNS and 0 to 2 wt % for BNNT using a Speed Mixer DAC 600.2 VAC-P under vacuum. The mixture was then poured into a silicone rubber mold and left to cure overnight at room temperature. The composite samples were then hot-pressed at a pressure of 10 tons and a temperature of 60 °C followed by a curing cycle of 80 °C for 1 h and 140 °C for 8 h. After curing, the samples were removed from the mold and polished to remove any marks and surface voids. It was observed that the viscosity of the epoxy-BN mixture increases significantly with increasing BN content and becomes a very stiff paste at a high BN content. This effect is particularly pronounced for the smallest BN particles (5 μm), for which the 40% BN content could not be achieved because the surface area to volume ratio increases as the particle size decreases. Additionally, a higher viscosity was observed in an epoxy 2–30 wt % BNNS mixture with 2 wt % BNNT loading compared to the low viscous epoxy 1–30 wt % BNNS mixture at the same loading, attributed to the inherently higher viscosity of epoxy 2.

### Characterization

The morphology and microstructure of the materials were studied by using field emission scanning electron microscopy (FESEM) on a TESCAN MIRA3. The loading of fillers and thermal stability of the composites was determined through thermogravimetric analysis (TGA) using a simultaneous thermal analyzer (STA 449 F5 NETZSCH, Germany) in a nitrogen atmosphere with a heating rate of 10 °C/min. Thermal conductivity (*k*) was calculated using the equation *k* = *D*·Cp·*d*, where *D* is the thermal diffusivity measured by laser flash apparatus (NETZSCH, LFA 427) for the through-plane direction and a homemade IR thermoreflectance instrument for the in-plane direction. Cp is the specific heat capacity determined by differential scanning calorimetry (DSC Q100, TA Instruments) with a heating rate of 3 °C/min under a nitrogen atmosphere, and *d* is the density that was determined by the Archimedes method. The relative densities of all samples were greater than 99%, and the uncertainties in Cp and *D* were 2 and 5%, respectively.

## Results and Discussion

**Figure 2:**
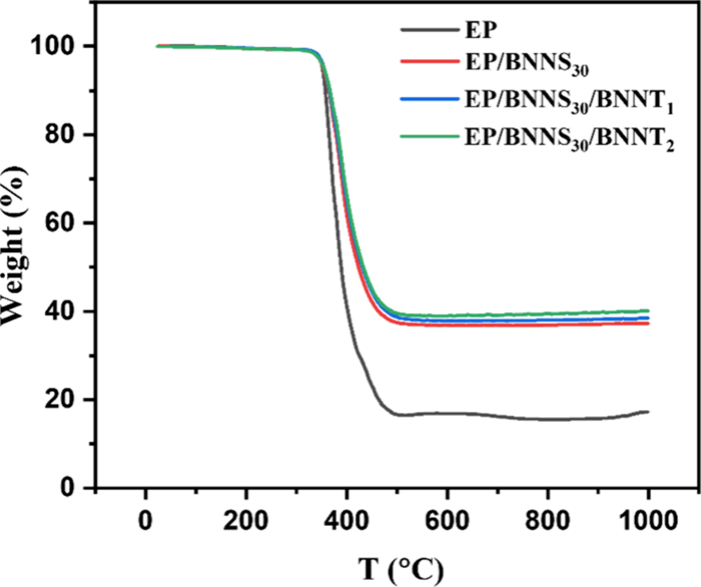
TGA curves of the neat epoxy and EP1/BNNS/BNNT composites.

The performance of the epoxy/hybrid nanofiller composites was evaluated in terms of thermal conductivity and stability. The thermal stability of the composites was determined by TGA experiments under an inert atmosphere, as discussed in the Materials and Methods section. The TGA curves of the pristine epoxy 1 and hybrid filler/epoxy 1 composites showed similar thermal degradation trends around 395 °C that match with literature,^18^ as shown in Figure 2. The addition of BNNS and BNNT slightly improves the thermal stability of the composite and retards the thermal degradation process up to 2–3%, and the higher the filler content, the better the thermal stability. The improvement in thermal stability of the composite is mainly attributed to the fact that the BNNS and BNNT, which are uniformly dispersed in the epoxy, act as a heat-resistant layer and mass transfer barrier to hinder the thermal degradation process.^18^

The thermal conductivity of the composite samples was calculated by multiplying the measured Cp, *D*, and mass density (*d*) values. Both the in-plane and out-of-plane thermal diffusivities of the composites containing BNNS and BNNT as a function of filler loadings were measured by the IR thermoreflectance method and the laser flash method, respectively, and are shown in Figure S1. The thermal diffusivity of the samples was measured at five different locations at 30 °C using the IR thermoreflectance method, and more information on the measurements can be found in the Supporting Information. The specific heat capacity measurements are shown in Figure S1. Our analysis revealed that the inclusion of both CNTs and graphene resulted in changes in the Cp of the composites relative to the reference composite (LY564, 30 wt % BNNS). These variations in Cp can be attributed to the unique thermal properties of the fillers and their interactions with the epoxy matrix.

**Figure 3:**
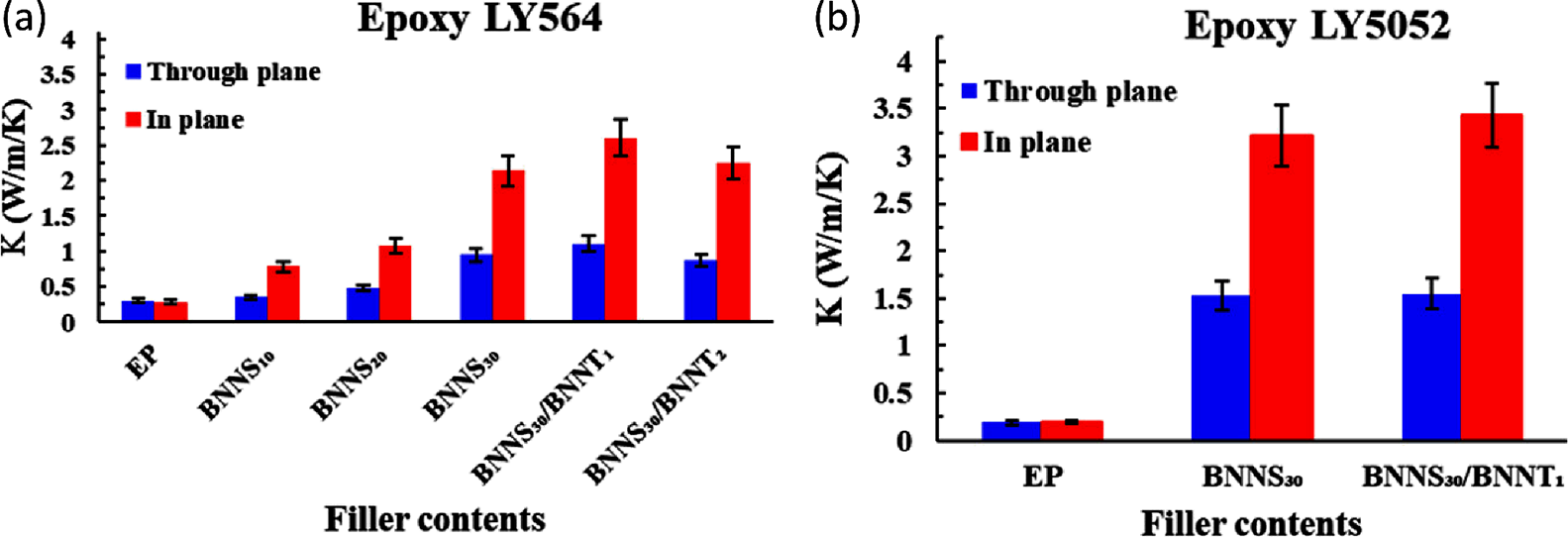
Thermal conductivities of EP and BNNT/BNNS/EP composites at different filler contents for different epoxies: (a) Araldite LY564 and (b) Araldite LY5052.

To investigate the effect on the thermal conductivity, Figure 3 compares the thermal conductivities of the composites containing BNNS and BNNT as a function of filler loadings. Both in-plane and out-of-plane directions exhibit anisotropic thermal conductivity due to the orientation of the BNNS filler. Oriented fillers form a thermal conduction path along the in-plane direction, resulting in a higher in-plane thermal conductivity than the out-of-plane thermal conductivity.^19^ It is observed that the thermal conductivities of all the composites measured increase along with the filler loadings. The thermal conductivities of the neat unfilled epoxies LY564 and LY5052 are 0.27 and 0.2 Wm^–1^ K^–1^, respectively.

With 1 wt % BNNT incorporated into the 30 wt % BNNS-filled epoxy composites, they possess thermal conductivities of 2.6 and 3.4 Wm^–1^ K^–1^, i.e., a 10- to 17-fold increase in comparison to the pristine epoxy (Figure 3b). The increased thermal conductivity in (BNNT-BNNS)/epoxy composites is due to two factors; (i) higher thermal conductivity of BNNSs (these can more effectively improve the thermal conductivity of the composite) and (ii) BNNT/BNNS hybrid nanofillers are uniformly dispersed in the epoxy matrix, which helps the synergistic improvement of BNNT/BNNS hybrids on the thermal transport properties. This is because the bridging interactions between BNNTs and BNNSs can lead to the formation of a highly efficient 3D network that can form rather easily a path for the heat flow through the composites.^20^

**Figure 4:**
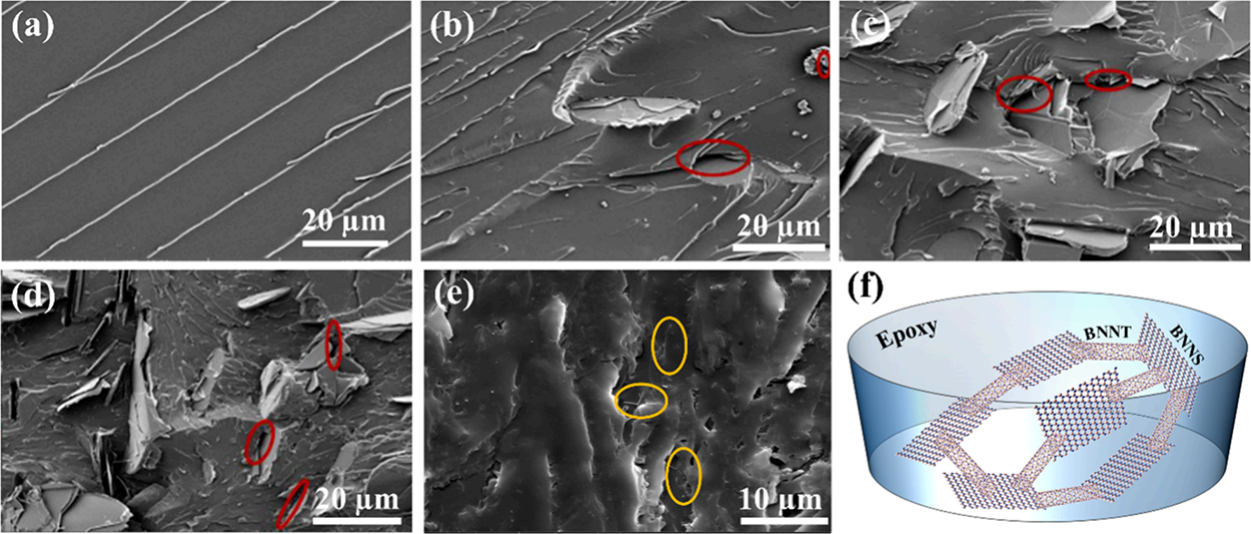
SEM images of the epoxy composites with different filler contents: (a) pristine epoxy 1, (b) EP1/BNNS_10_, (c) EP1/BNNS_20_, (d) EP1/BNNS_30_, and (e) EP1/BNNS_30_/BNNT_1_. (f) Schematic to show how heat is dissipated between BNNSs and BNNTs.

To better understand the improvement in thermal conductivity, we analyzed the structure of the composites to relate the performance to the distribution and alignment of the fillers. It was observed from the SEM images shown in Figure 4a–e, as well as the combined schematic illustration in Figure 4f, that most BNNTs are located in between BNNSs, which significantly increases the amount of contact area. This is because the contact geometry changes from a 2D point contact to a 1D linear contact compared with individual BNNS and BNNT nanofillers. Large contact areas tend to decrease the interfacial thermal resistance between the nanofillers and the epoxy matrix. This is because long and convoluted BNNT networks facilitate the connection of adjacent BNNSs, resulting in a highly effective thermal network and simultaneously low interfacial thermal resistance through increasing nanofillers/matrix contact area.^20^ However, at contents higher than 1 wt %, BNNTs were not effective in improving the thermal conductivity of 30 wt % BNNSs/epoxy composites due to increased phonon scattering density by internanotube junctions.^21^ The morphology and size of BNNS and BNNT nanofillers are shown in Figure S2.

The efficiency of the fillers in thermally conductive materials was characterized by the thermal conductivity enhancement (TCE), which is defined as
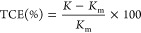
1where *K* is the thermal conductivity of the composite and *K*_m_ is the thermal conductivity of the matrix material. Table 1 summarizes some recent results from the literature on the thermal conductivity of various epoxy composites that used BN-based fillers. Based on our understanding, the thermal conductivity value obtained in this study for the epoxy composites with a BN-based hybrid filler content is higher than the previously reported values. We believe that this is due to the BNNSs acting as the primary pathways for thermal conductivity within the composites, with the BNNTs facilitating connections between the BNNSs. Furthermore, the method used in this study is comparatively straightforward and efficient when compared to modifying the BN surface using strong acids or bases.

**Table 1:** Thermal Conductivity Values of Epoxy Composites with Different BN-Based Fillers

types of epoxy	fillers	filler fraction	*K* (W/m/K)	TCE (%)	year and ref
not revealed	PBS-(BNNT + BNNS)	2 wt % (BNNT + BNNS)	0.47	135	2013 (^20^)
Araldite LY564 and LY5052	BNNS/BNNT	30/1 wt % BNNT	3.43 and 2.6	1705 and 940	**this work**
DGEBA (EPIKOTE)	BN/CNT	40 wt % BN/1 wt % CNT	1.1	450	2019 (^22^)
cycloaliphatic	m(BN/CNT)	10 wt % BN/2 wt % CNT	0.63	215	2020 (^23^)
bisphenol A diglycidyl ether (E-51)	BNNS/MWCNTs@mSIO_2_	20 wt % BNNS/0.1 MWCNTs@mSIO_2_	0.68	240	2021 (^24^)
dignycidyl ether of bisphenol A (DGEBA) epoxy NPEL-12	BN/MWCNTs	30 vol % BN vol % MWCNT	1.91	855	2011 (^25^)
3-glycidoxypropyltrimeth oxysilane	BNNS/MWCNT/SiO2@KH 560	10 wt % BNNS/0.5 wt % MWCNTs/0.5 wt % SiO2	0.93	365	2022 (^26^)

## Conclusions

The interaction between the 1D and 2D fillers allowed the hybrid composites to exhibit greatly enhanced in-plane thermal conductivities of 2.6 and 3.4 Wm^–1^ K^–1^, respectively, which are 10–17 times higher than the thermal conductivity of the pristine epoxy, when the BNNS/BNNT volume fractions are 30 and 1% in epoxies LY564 and LY5052, respectively. The significant enhancement in thermal conductivity observed in our epoxy composites can be attributed to the synergistic interaction between BNNT and BNNS hybrids, which facilitates the formation of an efficient three-dimensional (3D) heat conduction network within the matrix. The underlying mechanisms behind this can be attributed to several different factors as follows.

### Interfacial Contact Area Enhancement

The incorporation of BNNTs between BNNS layers increases the contact area between the fillers and the epoxy matrix. This increased contact area facilitates better phonon transport across the filler–matrix interface, reducing interfacial thermal resistance and improving overall thermal conductivity.

### Bridging Effect of BNNTs

The high aspect ratio of BNNTs and their ability to form bridges between adjacent BNNS layers creates continuous pathways for heat conduction. This bridging effect ensures efficient phonon transport across the composite, further enhancing the thermal conductivity.

### Synergy

The combination of 1D (BNNTs) and 2D (BNNSs) fillers in the epoxy matrix results in a synergistic effect on the thermal conductivity improvement. The 1D-2D hybrid structure allows for the creation of an interconnected thermal network that facilitates rapid and efficient heat transfer throughout the composite.

### Reduced Phonon Scattering

The well-dispersed BNNTs and BNNSs in the epoxy matrix minimize phonon scattering, allowing for smoother phonon transport pathways. This reduction in the phonon scattering density contributes to the observed enhancement in thermal conductivity.

In summary, the improved thermal conductivity in our epoxy composites can be attributed to the combined effects of increased interfacial contact area, the bridging effect of BNNTs, synergistic interactions between BNNTs and BNNSs, and reduced phonon scattering. These factors collectively contribute to the formation of an efficient 3D heat conduction network. This research provides new insights into the design, fabrication, and improvement strategies for polymer-based composites with excellent thermal conductivity. The BNNSs/BNNTs/epoxy composites have potential applications in advanced electronic packaging technology such as thermal interface materials, underfill materials, molding compounds, and flexible substrates.

## Supporting Information Available

In-plane and through-plane thermal diffusivity measurement by IR thermoreflectance and laser flash setups (PDF)
