# Supplementary material for: Synergistic Improvement in the Thermal Conductivity of Hybrid Boron Nitride Nanotube/Nanosheet Epoxy Composites
Source: ACS Appl Nano Mater. 2024 May 20;7(11):13142–6. doi: 10.1021/acsanm.4c01646 (PMC11190995; doi:10.1021/acsanm.4c01646)
Supplement: Supplementary file 1 — an4c01646_si_001.pdf [file an4c01646_si_001.pdf]

Supporting Information

Synergistic Improvement in the Thermal Conductivity of Hybrid Boron Nitride Nanotube/Nanosheet Epoxy Composites

Rajeshkumar Mohanraman, Ian A. Kinloch, Pietro Steiner, Coskun Kocabas, Mark A. Bissett*

Department of Materials, Henry Royce Institute, National Graphene Institute, University of Manchester, Manchester, UK, M13 9PL

E-mail: Mark.Bissett@Manchester.ac.uk

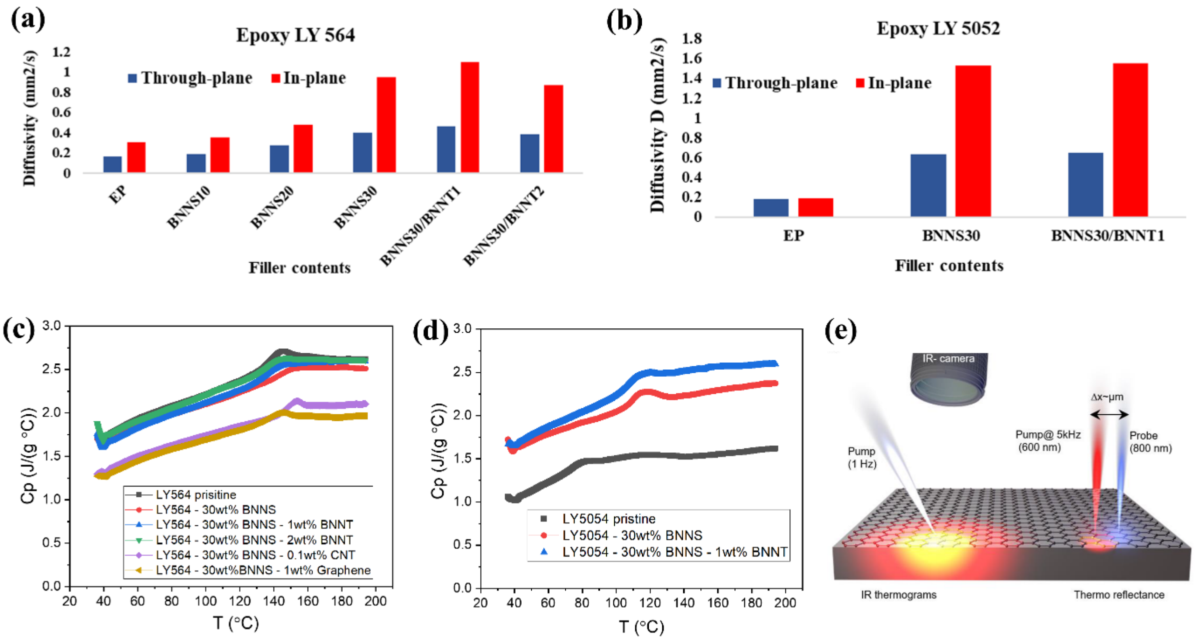

Fig S1. (a)-(d) Thermal diffusivity D and specific heat capacity Cp of EP and BNNT/BNNS/EP composites at different filler contents for different epoxy's Araldite LY 564 and Araldite LY 5052 and (e) a schematic illustration showing the use of thermoreflectance and infrared thermograms for thermal characterization at micrometer and millimeter length scales.

In plane and through plane thermal diffusivity measurement by IR thermoreflectance and laser flash apparatus respectively are shown in Figure S1a and b. Temperature dependence of the specific heat capacity, Cp of epoxy-based composites are shown in Figure S1c and d. We employed infrared thermography and modulated thermoreflectance techniques to measure the

in-plane thermal conductivity of epoxy-based composites shown in Figure S1e. The thermoreflectance method involves modulating a 600nm pump laser at 5kHz and measuring the thermal wave profile by moving an 800nm probe laser. IR thermography, on the other hand, records the propagation of heat waves generated by a supercontinuum laser modulated at 1Hz using a camera.1

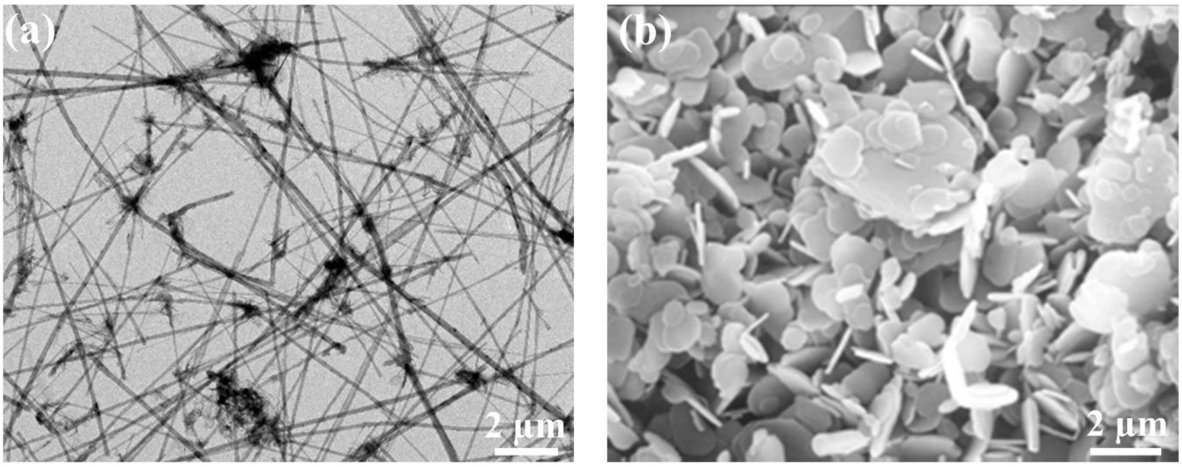

Fig S2. SEM images of as received nanofillers (a) Boron Nitride nanotubes and (b) h-BN nanoplatelet powder respectively.

Figure **S2** showcases the morphology and size distribution of boron nitride nanotubes (BNNT) and hexagonal boron nitride (hBN) platelets utilized in this study. The BNNTs display a length greater than 10 µm, while the hBN platelets have an average diameter of approximately 5 µm. These size parameters are identified as pivotal for achieving effective thermal alignment within the epoxy composites.

References

1. C. Kocabus et al, ‘‘Electrically controlled heat transport in multilayer graphene’’. DOI: 10.48550/arXiv.2202.10342
